# Molecular Detection and Genotyping of *Chlamydia psittaci* in Captive Psittacines from Costa Rica

**DOI:** 10.1155/2013/142962

**Published:** 2013-09-18

**Authors:** Jessica Sheleby-Elías, Ántony Solórzano-Morales, Juan José Romero-Zuñiga, Gaby Dolz

**Affiliations:** ^1^Maestría en Enfermedades Tropicales, Posgrado Regional en Ciencias Veterinarias Tropicales, Escuela de Medicina Veterinaria, Universidad Nacional, P.O. Box 86, 3000 Heredia, Costa Rica; ^2^Laboratorio de Entomología y Medicina Poblacional, Programa MEDPOB, Escuela de Medicina Veterinaria, Universidad Nacional, P.O. Box 86, 3000 Heredia, Costa Rica

## Abstract

Oropharyngeal and cloacal swabs from 117 captive psittacine birds presented at veterinary clinics (88) and from shelters/rescue centers of wildlife (29) were collected to determine the prevalence of *C. psittaci* in captive birds in Costa Rica. Samples were collected during 2009 from a total of 19 different species of parrots, with *Ara macao* (33), *Amazona autumnalis* (24), *Amazona ochrocephala* (21), and *Ara ararauna* (8) being the most representative species sampled. *C. psittaci* was detected in four (3.4%) birds using molecular detection (PCR). The positive samples belonged to birds presented at veterinary clinics; three of them were *Ara macao* and one *Amazona ochrocephala*. Three birds were adults; all positive birds showed no symptoms of illness and lived in homes with other birds, two in San José and two in Heredia. Sequencing was used to confirm the PCR positive results, showing that two samples of *C. psittaci* belonged to genotype A, representing the first report of the presence of this genotype in Costa Rica. The detection of this bacterium in captive psittacine birds shows that there is a potential risk for people living or having contact with them and that there is a possibility of infecting other birds.

## 1. Introduction

Avian chlamydiosis is caused by *Chlamydia psittaci*, a Gram-negative, intracellular bacterium, with nine known genotypes (A–F, E/B, M56, and WC) [1]. *C. psittaci* has been detected in 465 species of birds [2], but the highest infection rates are found in parrots (*Psittacidae*) and pigeons (*Columbiformes*). In parrots, the prevalence varies between 16% and 81% [3–5]. Progression to clinical infection is dependent on the nature of the infecting strain and on host species. Avirulent strains generally produce asymptomatic infections in adult birds, and these may excrete the organism for several months. Large quantities of the agent can be found regularly or intermittently in feces, lacrimal fluids, nasal discharges, oropharyngeal mucus, and crop milk of infected birds [6]. 

Prolonged and subacute clinical forms are common. Extreme environmental changes or concurrent infections may cause the onset of clinical disease. Avian chlamydiosis presents from nonspecific clinical signs to acute systemic illness, latter especially in young animals; lethargy, anorexia, dehydration, depression, hyperthermia, nasal and ocular discharges, abnormal excretions, and greenish diarrhea are mainly reported [6, 7].

All *C. psittaci* genotypes can be transmitted to humans where they also cause a disease called psittacosis or parrot fever [7]. This transmission may occur by inhalation or direct contact with infected birds [8]. In humans the disease may vary from nonspecific symptoms similar to flu to severe pneumonia. Cases of endocarditis and encephalitis have also been attributed to this bacterium [9].

Diagnosis is made using a range of techniques: serological techniques include immunofluorescence, complement fixation, and immunoenzymatic assays. The main disadvantage of serological techniques is that asymptomatic birds, which still excrete the agent, generally show low antibody titers. In addition, the detection of antibodies in birds only suggests exposure to the agent; it is not possible using this approach to determine whether the birds are active carriers of the bacterium or have cleared a previous infection [8]. Among direct techniques the isolation of the bacterium by culture on McCoy, Buffalo Green Monkey, or chicken cell lines is considered the golden standard for chlamydiosis diagnosis [10] the advantage is its sensitivity, since it is possible to detect a small number of microorganisms after two or three passages [6]. The disadvantage is that it requires a Biosecurity Level 3 Laboratory. Because many birds are subclinical carriers and *Chlamydia* organisms are excreted intermittently in feces and other secretions, a single sample analysis may give false negative results [11, 12]. 

Direct immunoenzymatic tests are also very useful; they reported a high specificity; however at least 600 elementary bodies are needed in the sample, to avoid false negative results [13, 14]. Polymerase chain reaction (PCR) represents a specific, sensitive, and quick technique to detect *C. psittaci* [15]. Particularly the nested PCR developed by Kaltenböck et al. [16] has shown higher sensitivity than other protocols [17]. As before, the disadvantage of direct detection techniques is, that the agent is excreted intermittently [11] consequently collection of multiple samples over two to three days is recommended. Therefore, only a positive result through a direct technique is 100% reliable for the diagnosis of *C. psittaci*. Finally, birds that have begun treatment with antibiotics may show false negative results because the infection level has fallen below detection limits [18]. If the treatment protocol is not carried out as indicated (4 or 6 weeks depending on selected antibiotic and dose) the bird can excrete again the bacteria.

A study conducted in 2001 in Costa Rica detected antibodies against *C. psittaci *in 12.39% of 129 macaws (*Ara macao* and *Ara ambigua*) in captivity using an ELISA [19]. The objective of the present study was to detect the presence and to characterize *C. psittaci* using molecular techniques (PCR and sequencing) in psittacine birds in captivity of Costa Rica.

## 2. Materials and Methods

### 2.1. Size, Type of Sample, and Data Collected from the Analyzed Birds

Approximately 140.200 parrots and parakeets were estimated to live illegally in captivity in households in Costa Rica [20]. To determine the presence of *C. psittaci*, the formula described by Wayne [21] was used, with an expected prevalence of 7% (95% confidence level and a 5% error). A sample size of 101 birds to be analyzed was determined. Expected prevalence of 7% was used, because seroprevalence generally overestimates real prevalence, since present and past infections are measured, and due to the fact that the possibility of finding antibodies in a population is higher than that of finding antigens.

A total of 117 samples from birds were collected in 2009 from veterinary clinics from the Central Valley (*n* = 88) and from shelters/rescue centers (*n* = 29) from different provinces of Costa Rica (Table 1). These birds lived for unknown time illegally in private households and were presented to veterinary clinics for routine examination or were kept for some time in shelters/rescue centers. Cloacal and oropharyngeal swab samples were taken from each bird and preserved for a maximum of five days at 4°C; once in the laboratory these swabs were kept at −20°C until DNA extraction and molecular analysis was performed. In addition, a clinical survey was carried out, to collect the following data of the birds: species, province of origin, age, and symptoms related to chlamydiosis (loss of appetite, weight loss, ocular or nasal secretions, greenish/yellowish watery stool, and neurological problems). The survey included also questions about the health status, especially about respiratory symptoms prior to the sampling data of owners, their families, or workers. The distribution of the psittacines analyzed in the present study by species is presented in Table 2.

### 2.2. Polymerase Chain Reaction (PCR) for *C. psittaci*

For DNA extraction DNeasy Blood & Tissue Kit of QIAGEN was used, proceeding according to the manufacturer's instructions. For the detection of *C. psittaci* a nested PCR described by Kaltenböck et al. [16] and modified by Theegarten et al. [22] was used, which amplifies partially gene *ompA* (outer membrane protein A) to identify the genus *Chlamydia* spp. The primers used were 191CHOMP (5′-GCI YTI TGG GAR TGY GGI TGY GCI AC-3′) and CHOMP371 (5′-TTA GAA IC [GT] GAA TTG IGC [AG] [TC] IA GTG IGC IGC TT-3′). Reactions with 18.9 *μ*L Dream Taq PCR Master Mix 2X (Fermentas), 1.0 *μ*L of each primer (0.1 *μ*M), 0.5 *μ*L DNA (~20 *μ*g), and 4.6 *μ*L water (molecular biology grade, Fermentas) were prepared to a final volume of 25 *μ*L. Amplification protocol consisted of an initial denaturation at 95°C for 30 s, followed by 35 cycles of denaturation (95°C for 30 s), alignment (50°C for 30 s), extension (72°C for 30 s), and a final extension at 72°C for 7 min. PCR products were visualized by agarose gel electrophoresis (1.4%) in TBE (Tris Base, boric acid, EDTA, pH8, 0.5 M), stained with ethidium bromide (0.5 *μ*g/mL). GeneRuler 100 bp DNA Ladder Plus (Sm0321, Fermentas) was used as marker. Samples that show bands with weights 576–597 pb were considered positive. All amplification products were subjected to a second PCR to identify *C. psittaci*, using the primers CHOMP 336 s (5′-CCR CAA TTT CTR GAY TTC AWY TTG TTR en GMT-3′) and 218PSITT (5′-GTA ATT TCI AGC CCA GCA CAA TTY GTG-3′), in a reaction with proportions of reactants as described above, varying the conditions of the cycler: 95°C for 30 s followed by 20 cycles of 95°C for 30 s, 60°C for 30 s, 72°C for 30 s, and 72°C for 7 min. The second PCR products were visualized by agarose gel electrophoresis as described above. Samples that show bands with weights 389–404 bp were considered positive. DNA of *C. psittaci* donated by the Clinic of Birds, Reptiles, Amphibians and Fish, Justus Liebig University, Giessen, Germany, was used as positive control; water (molecular biology grade, Fermentas) was used as negative control.

### 2.3. Sequencing, Genotyping, and Phylogenetic Tree

Samples positive by nested PCR were genotyped through analysis of *ompA* gene sequences [23]. The primers used were CPsittGenoFor (5′-GCTACGGGTTCCGCTCT-3′) and CPsittGenoRev (5′-TTTGTTGATYTGAATCGAAGC-3′), which amplify conserved regions of the *ompA* gene covering four variable domains [23]. The volume of the reaction (25 *μ*L) included 12.5 *μ*L Dream TaqTM PCR Master Mix 2X (Fermentas), 1.0 *μ*L of each primer (20 pmol/*μ*L), 5 *μ*L DNA (~20 *μ*g), and 5.5 *μ*L of water (molecular biology grade, Fermentas). Steps for amplification consisted of an initial denaturation at 95°C for 10 minutes, 35 cycles of 95°C for 1 min, 55°C for 1 min, 72°C for 1 min, and a final extension at 72°C for 10 min. The size of the amplified fragment was 1041 bp, visualized in electrophoresis as described above. PCR products were purified using the QIAquick (QIAGEN) kit, proceeding according to the manufacturer's instructions.

Positive samples were sent to Macrogen (Seoul, Korea) for sequencing. Partial sequences were aligned with BioEdit Sequence Alignment Editor [24] and compared using the BLASTn algorithm with the database of NCBI (National Center for Biotechnology Information). Afterwards they were imported in MEGA 5 [25] for the design of the phylogenetic tree. The evolutionary history was inferred using the UPGMA method [26]. The percentage of replicate trees in which the associated taxa clustered together in the bootstrap test (10000 replicates) was shown next to the branches [27]. The tree was drawn to scale, with branch lengths in the same units as those of the evolutionary distances used to infer the phylogenetic tree. The evolutionary distances were computed using the Jukes-Cantor method [28] and were shown in the units of the number of base substitutions per site. The analysis involved 12 nucleotide sequences. Reference sequences of the nine *C. psittaci* genotypes available at the database of GenBank, A (accession number AY762608), B (AF269265), C (L25436), D (AF269266), E (X12647), F (AF269259), E/B (AY762613), M56 (AF269268), and WC (AF269269), were included in the analysis [29]. The phylogenetic tree was compared with the ompA sequence of *Chlamydia caviae* (GPIC, GenBank AF269282) [30].

## 3. Results

A total of four (3.4%) out of 117 analyzed psittacines were found to be positive to *C. psittaci*. All positive birds had attended veterinary clinics for routine exams, with three of them belonging to the species *Ara macao* and one to *A. ochrocephala*; none showed symptoms related to the disease. In two birds DNA of *C. psittaci* was detected in the oropharyngeal swabs, and in other two birds in cloacal swabs, none of them presented DNA of the agent in both types of swabs. Two birds were from San Jose and the other two from Heredia; only one of them was juvenile, while the other three were adult. In all cases, the birds lived in households, and with other birds, that were not sampled in this study (Table 3). 

**Figure 1:**
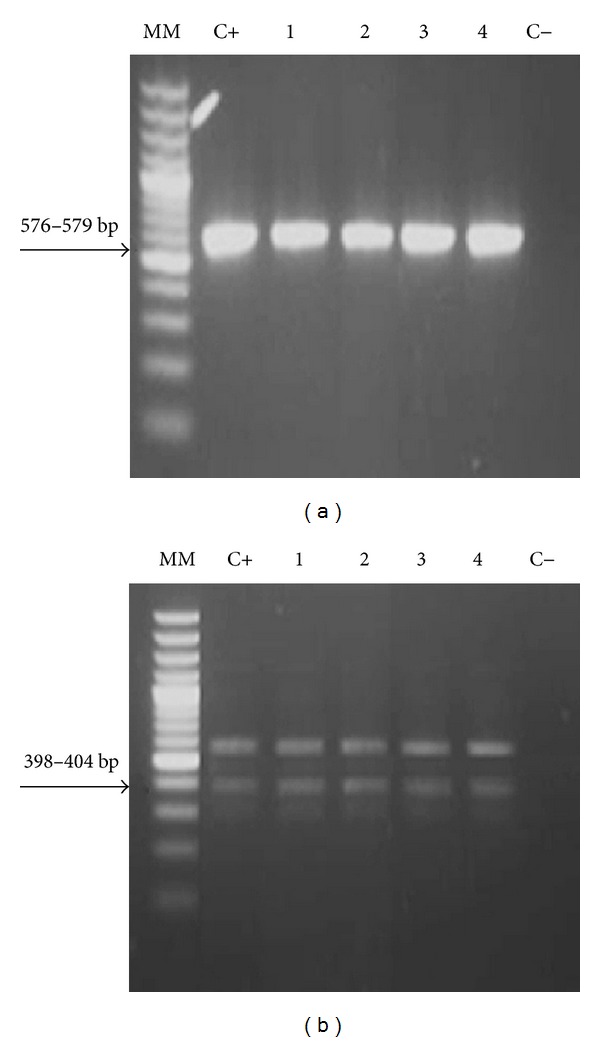
Gel electrophoresis of PCR products of *Chlamydia* spp. (a) and *C. psittaci *(b). (MM: molecular marker; C+: positive control; 1–4: Birds 1, 2, 3, and 4, positive to *Chlamydia* spp. and *C. psittaci*; C−: negative control).

**Figure 2:**
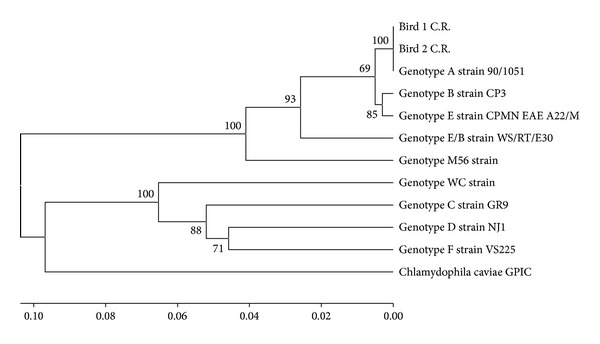
Phylogenetic tree of gene *ompA* sequence of two samples of psittacines (Bird 1 C.R. and Bird 2 C.R.) of Costa Rica positive to *C. psittaci*.

Only four samples yielded positive results in the first PCR (Figure 1(a)) and amplified afterwards the specific band (389–304) in the nested PCR; however, remaining DNA product from the first PCR was also detected (Figure 1(b)). The sequencing was carried out only with two positive samples (Birds 1 and 2) confirming the results and identifying these as *C. psittaci* genotype A (Figure 2). BLASTn analysis resulted in a 100% nucleotide identity between both samples, 100% sequence homology with the 90/1051 strain (GenBank AY762608), and 99% with other genotype A strains [84–55 (Y16561), 6BC (X56980), and VS1 MN Zhang (AF269281)]. DNA from the other two birds (3 and 4) was not sufficient to carry out sequentiation analysis. 

## 4. Discussion

This study reports for the first time the presence of the bacterium *C. psittaci* in parrots in captivity of Costa Rica. Although a seropositivity of 12.4% was previously reported in *Ara macao* and *Ara ambigua*, using an indirect ELISA technique [19], detection and genotyping of the agent using molecular techniques (PCR and sequencing) had not been carried out to date, either in animals or in humans in Costa Rica and Central America. 

The low positivity determined using this PCR-based detection (3.4%) was not expected, taking into account the results obtained by Herrera et al. [19] and others [3–5]. This can be explained by the different techniques used and the higher possibility of finding antibodies than that of directly detecting the causative organism in a population, which is known to be excreted in infected birds only intermittently through feces or oropharyngeal secretions into the environment. It is possible that, if in the present study the samples had been taken consecutively for 2 to 3 days, a higher percentage of positive birds would had been found [31].

It is important to stress out, however, that the birds detected positive were presented to veterinary clinics for routine examination and were not showing clinical symptoms, which is consistent with reports from Gerlach [6], who found that birds may have asymptomatic infections for various reasons, whether they were infected with a low virulence strain or due to resistance of some bird species. Thus, avirulent strains produce generally asymptomatic infections in adult birds, and these may excrete the organism for several months. Factors that can influence the development of clinical symptoms are conditions in which the birds are kept; good management conditions reduce stressful situations, while extreme environmental changes or concurrent infections can cause the onset of clinical disease. Finally, prolonged subacute forms without clinical signs are also common [6]. 

The sequencing of the positive samples confirmed the molecular diagnosis from two parrots. In addition, determining the presence of *C. psittaci* genotype A in psittacines in captivity of Costa Rica agrees widely with the literature reports that A genotype is usually found in parrots, which is also the most common genotype identified in humans [32–34]. 

The results show the importance of the diagnosis of *C. psittaci* by PCR in birds, when they are introduced to a shelter, rescue center, Zoo, or other condition of captivity, to determine whether they are carriers and possible source of infection to other birds and, on the other hand, whether they are a risk to people that are in contact with these birds (owners and veterinarians, among others). In this respect, none of the owners of the *C. psittaci* positive birds nor their families or workers reported respiratory symptoms in the survey. According to Drews [20], parrots represent 80% of the wild animals in captivity in Costa Rica. Based on the results obtained in this research it is important to alert the authorities and the general population. The possession of wild birds is illegal in Costa Rica according to the Conservation Law of Wildlife [35]; also it represents a risk to public health, since all samples identified as positive to *C. psittaci *came from parrots that lived in homes. 

We recommend including the molecular diagnosis of *C. psittaci* in humans, especially in those people who are in contact or who live with parrots, as well as performing prevalence studies in other birds, such as *Columbiformes* and especially free living psittacines, to determine whether it poses a serious problem for wildlife of Costa Rica. Ethical guidelines were applied during this investigation, since live wild animals were manipulated.

## Figures and Tables

**Table 1:** Distribution of analyzed psittacines by province.

Province	VC^2^	Shelters^3^	Total
Alajuela	11	1	12
Puntarenas	1	11	12
Guanacaste	—	8	8
Cartago	5	9	14
San José	49	—	49
Heredia	15	—	15
Limón	2	—	2
NR^1^	5	—	5

Total	88	29	117

^1^NR: not reported; ^2^VC: birds submitted to veterinary clinics. ^3^Shelters: birds from shelters or rescue centers.

**Table 2:** Distribution of analyzed psittacines by species.

Species	Common name	VC^1^	Shelters^2^	Total
*Ara ambigua *	Lapa verde	1	1	2
*Ara macao *	Lapa roja	22	11	33
*Amazona auropalliata *	Loro nuca amarilla	3	3	6
*Amazona autumnalis *	Loro frente roja	17	7	24
*Aratinga finschi *	Perico frente rojo	5	—	5
*Amazona aestiva *	Loro frente azul	1	—	1
*Amazona ochrocephala *	Loro frente amarilla	17	4	21
*Amazona farinosa *	Loro cabeza verde	2	1	3
*Brotogeris jugularis *	Chucuyo	1	1	2
*Psittacus erithacus *	Loro gris	1	—	1
*Amazona oratrix *	Loro cabeza amarilla	1	—	1
*Ara ararauna *	Lapa azul y amarillo	8	—	8
*Ara militaris *	Guacamayo verde	1	—	1
*Pionus senilis *	Loro cabeza de viejo	2	—	2
*Cacatua alba *	Cacatúa blanca	1	—	1
*Aratinga nana *	Perico pecho oliva	1	—	1
*Amazona albifrons *	Loro frente blanco	2	1	3
*Ara hibrido*	Lapa hibrida	1	—	1
*Agapornis sp. *	Periquito de amor	1	—	1

Total		88	29	117

^1^VC: birds submitted to veterinary clinics. ^2^Shelters: birds from shelters or rescue centers.

**Table 3:** Description of birds positive to *C. psittaci. *

	Bird 1	Bird 2	Bird 3	Bird 4
Species	*A. macao *	*A. macao *	*A. macao *	*A. ochrocephala *
Province	San José	San José	Heredia	Heredia
Age	Juvenile	Adult	Adult	Adult
Symptoms	None	None	None	None
Positive swab	Oropharyngeal	Oropharyngeal	Cloacal	Cloacal
No. of birds living in the household	40	2	12	3
